# Receptor binding and structural basis of raccoon dog ACE2 binding to SARS-CoV-2 prototype and its variants

**DOI:** 10.1371/journal.ppat.1012713

**Published:** 2024-12-05

**Authors:** Chunliang Luo, Linjie Li, Yuhang Gu, Hangchuan Zhang, Zepeng Xu, Junqing Sun, Kaiyuan Shi, Sufang Ma, Wen-Xia Tian, Kefang Liu, George F. Gao

**Affiliations:** 1 College of Veterinary Medicine, Shanxi Agricultural University, Jinzhong, China; 2 CAS Key Laboratory of Pathogen Microbiology and Immunology, Institute of Microbiology, Chinese Academy of Sciences (CAS), Beijing, China; 3 Beijing Life Science Academy, Beijing, China; 4 School of Life Sciences, Yunnan University, Kunming, China; 5 Faculty of Health Sciences, University of Macau, Macau SAR, China; 6 Hubei Provincial Key Laboratory for Protection and Application of Special Plants in Wuling Area of China, College of Life Sciences, South-Central Minzu University, Wuhan, China; University of Hong Kong, HONG KONG

## Abstract

Raccoon dog was proposed as a potential host of SARS-CoV-2, but no evidence support such a notion. In our study, we investigated the binding affinities of raccoon dog ACE2 (rdACE2) to the spike (S) protein receptor binding domain (RBD) of SARS-CoV-2 prototype (PT) and its variants. It revealed that the binding affinities of RBD from SARS-CoV-2 variants were generally lower than that of the PT RBD. Through structural and functional analyses, we found amino acids H34 and M82 play pivotal roles in maintaining the binding affinity of ACE2 to different SARS-CoV-2 sub-variants. These results suggest that raccoon dogs exhibit lower susceptibility to SARS-CoV-2 compared to those animal species with a high prevalence of SARS-CoV-2 transmission.

## Author summary

In our study, we investigated the potential role of raccoon dogs as hosts for SARS-CoV-2 by examining the binding affinities between the spike (S) protein receptor binding domain (RBD) of SARS-CoV-2 prototype (PT) and its variants with the ACE2 receptor from raccoon dogs (rdACE2). Our findings revealed that the binding affinities of the RBD from most of SARS-CoV-2 variants to rdACE2 were generally lower compared to the PT RBD. Detailed structural and functional analyses identified two key amino acids, H34 and M82, which are crucial in preserving the binding affinity of ACE2 to different SARS-CoV-2 sub-variants. These results indicate that raccoon dogs have a lower susceptibility to SARS-CoV-2 compared to other animal species known for a high prevalence of SARS-CoV-2 transmission. This study provides valuable insights into the host range of SARS-CoV-2 and highlights the importance of specific amino acid residues in ACE2 for virus-receptor interactions. Understanding these interactions is essential for assessing the zoonotic potential of SARS-CoV-2 and implementing effective measures to prevent interspecies transmission of the virus.

## Introduction

To date, seven coronaviruses have been identified capable of infecting humans: hCoV-NL63, hCoV-229E, hCoV-OC43, hCoV-HKU1, SARS-CoV-1, MERS-CoV and SARS-CoV-2 [1–8]. Additionally, Hu-PDCoV and CCoV-HuPn-2018 have been found to cause sporadic infections in children [9, 10]. Almost all these coronaviruses originate from animals [11].

SARS-CoV-2 has been continuously evolving into new variants since its outbreak in the end of 2019[12]. The World Health Organization (WHO) has identified five variants of concern (VOCs): Alpha, Beta, Gamma, Delta and Omicron. Moreover, Omicron is undergoing further evolution into multiple sub-variants with distinct characteristics [13–16]. In the early stages of the pandemic, our group discovered the virus entry receptor and assessed the receptor binding of SARS-CoV-2. We raised concerns about its broad interspecies binding spectrum and found that the Omicron variant further expanded the host range in subsequent studies [17–20]. Consequently, numerous animals have been reported to be naturally infected by SARS-CoV-2, totaling 45 species including pets, livestock, captive animals, and wildlife (https://www.fao.org/animal-health/situation-updates/sars-cov-2-in-animals/en).

Receptor binding plays a critical role in SARS-CoV-2 infection. The receptor binding domain (RBD) of the spike (S) protein binds to its host receptor, angiotensin-converting enzyme 2 (ACE2), facilitating virus entry [20]. The structures of SARS-CoV-2 and its variants binding to ACE2 receptors from humans, intermediate horseshoe bats, big-eared horseshoe bats, cats, dogs, horses, rabbits, white-tailed deer, mink, fox, hippopotami, minke whale and sea lion have been elucidated [17,20–30]. Additionally, the binding affinities of ACE2 receptors from intermediate horseshoe bats, white-tailed deer, fox, rabbits and hippopotami to various SARS-CoV-2 variants were assessed [22,23,26,27,29]. However, the origin of SARS-CoV-2 is yet under debate, and further investigation is needed to explore potential hosts.

During the SARS-CoV-1 pandemic, the virus was detected in raccoon dogs, suggesting their role as one of the hosts for SARS-CoV-1 [31]. With the emergence of the SARS-CoV-2 pandemic, raccoon dogs have become important suspected hosts [32]. Animal infection experiments have demonstrated that raccoon dogs typically display mild clinical signs, with viral replication and tissue lesions predominantly found in the nasal conchae [32]. After surveillance for SARS-CoV-2 was reported, some scientists suspected raccoon dog is the natural host of SARS-CoV-2[33,34]. However, the environmental samples cannot sufficiently prove the evidence for animal infection. A study examining coronaviruses in wild animals during the early stages of the COVID-19 outbreak, including 15 raccoon dogs, did not detect SARS-CoV-2 [35]. Another research team collected blood, tissue and swab samples from 229 raccoons and 11 raccoon dogs in Germany between 2021 and 2022 [36]. Despite thorough analysis using both molecular biological and serological methods, no evidence of SARS-CoV-2 was found in these samples. Until now, there has been no evidence to suggest that raccoon dogs can naturally become infected with SARS-CoV-2.

Recently, the structure of chimeric SARS-CoV-2 RBD (containing the core structure from SARS-CoV-1 and receptor-binding motif (RBM) from SARS-CoV-2) with a chimeric raccoon dog ACE2 (rdACE2) (containing the core structure from human ACE2 (hACE2) and virus-binding motif (VBM) from rdACE2) was determined [37]. However, it’s worth noting that this construct is an artificial creation and may not fully reflect real interactions. In structural biology, it’s considered a best practice to rely on the original amino acid sequences, as any mutations, whether located on the binding motif or not, can potentially impact functionality. For instance, the R346T substitution, located far from the RBM of SARS-CoV-2 RBD, was found to enhance ACE2 binding only when interacting with residue R493 but not Q493, which occurred through a mechanism involving long-range conformational changes [38,39].

Herein, we evaluated the binding affinities of rdACE2 with RBDs of SARS-CoV-2 PT and its variants. To elucidate the molecular basis of rdACE2 binding to SARS-CoV-2 PT and Alpha RBDs, respectively, we obtained cryo-EM rdACE2/S complex structures.

## Results

### The rdACE2 exhibits decreased binding affinities with most of the SARS-CoV-2 variants, except for Alpha and Delta

The full-length amino acid identity between rdACE2 and hACE2 is 83.88%. Compared to hACE2, there is a deletion at residue 21 in rdACE2. To allow for a more accurate comparison, we adjusted the numbering of amino acid sequence position of rdACE2 according to hACE2 **(S1 Fig)**.

**Fig 1:**
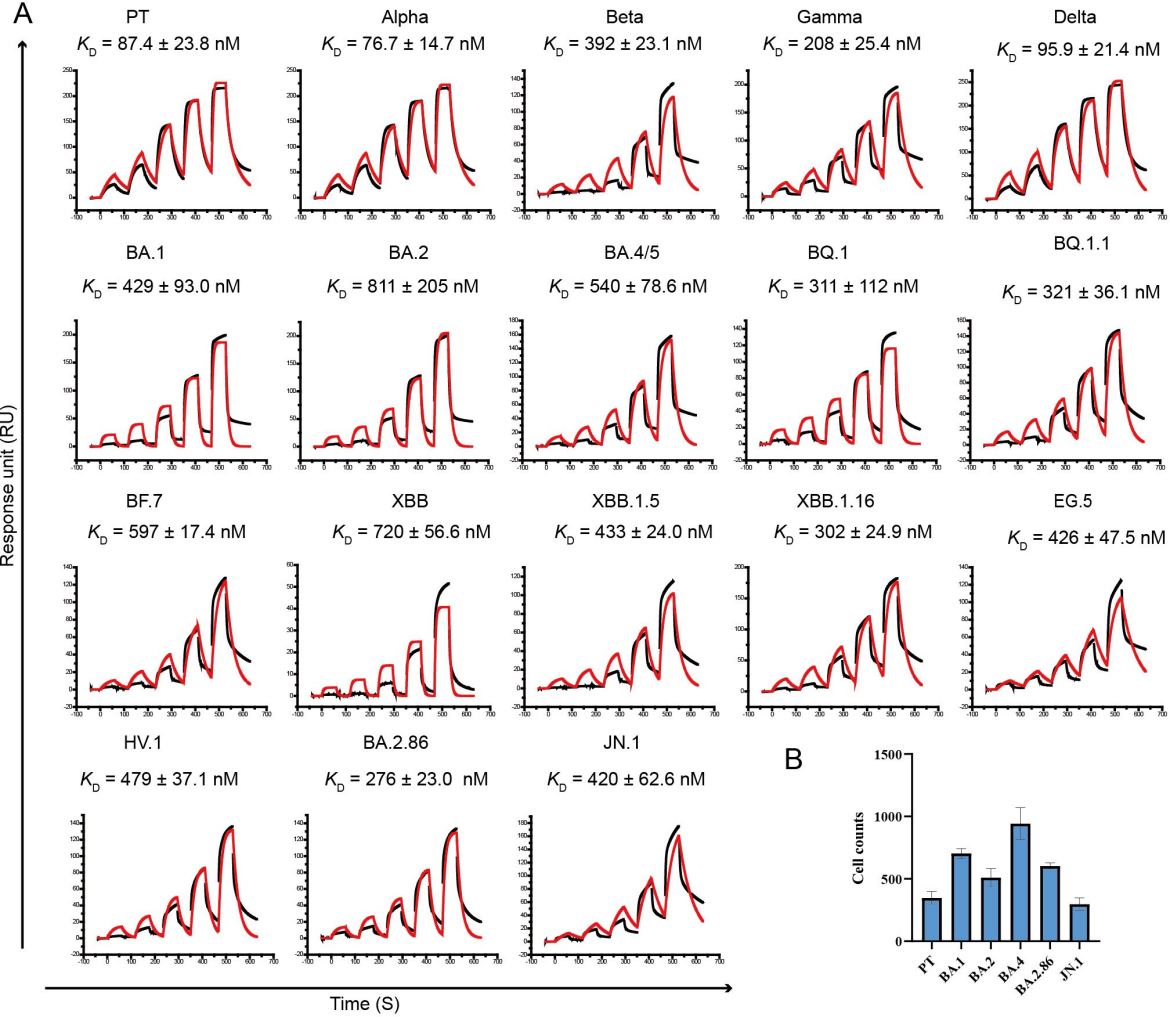
SPR assays for the binding of rdACE2 to RBDs from different SARS-CoV-2 variants and the infectivity of pseudotyped SARS-CoV-2 in BHK-21 cells transient expression of rdACE2. (A) Raw curves are represented by black lines, and fitted curves are represented by red lines. The dissociation constants (*K*_D_) are presented as mean ± SD calculated from three independent experiments. (B) Transduction of the pseudotyped SARS-CoV-2 PT and VOCs in BHK-21 cells transient expression of rdACE2. The data represent the results of three replicates.

To evaluate the binding affinities of rdACE2 to RBD of SARS-CoV-2 and its variants **(S2 Fig)**, surface plasmon resonance (SPR) experiments were conducted **(Figs 1A and S3)**. The results indicate that PT, Alpha and Delta variants exhibit comparable binding affinities to rdACE2 **(Fig 1A)**. On the other hand, Beta, Gamma and all the Omicron sub-variants (including BA.1, BA.2, BA.4/5, BQ.1, BQ.1.1, BF.7, XBB, XBB.1.5, XBB.1.16, EG.5, HV.1, BA.2.86 and JN.1) notably exhibit decreased binding affinities to rdACE2 **(Fig 1A)**. Particularly, the binding affinities of rdACE2 to the BA.2 RBD decreased by more than 10-fold, while the affinity to the XBB RBD dropped by nearly 10-fold **(Fig 1A)**.

To verify whether rdACE2 mediate SARS-CoV-2 VOCs entry, BHK-21 cells were transfected with plasmids containing enhanced green fluorescent protein (EGFP)-tagged rdACE2 and then infected by the vesicular stomatitis virus (VSV)-vectored pseudotyped viruses incorporating the S protein of SARS-CoV-2 VOCs. We found that all of the tested pseudotyped SARS-CoV-2, including PT, Omicron BA.1, BA.2, BA.4/5, BA.2.86 and JN.1, could invade into rdACE2-expressing BHK-21 cells **(Fig 1B)**.

### The overall structures of rdACE2 in complex with PT and Alpha S proteins

**Fig 2:**
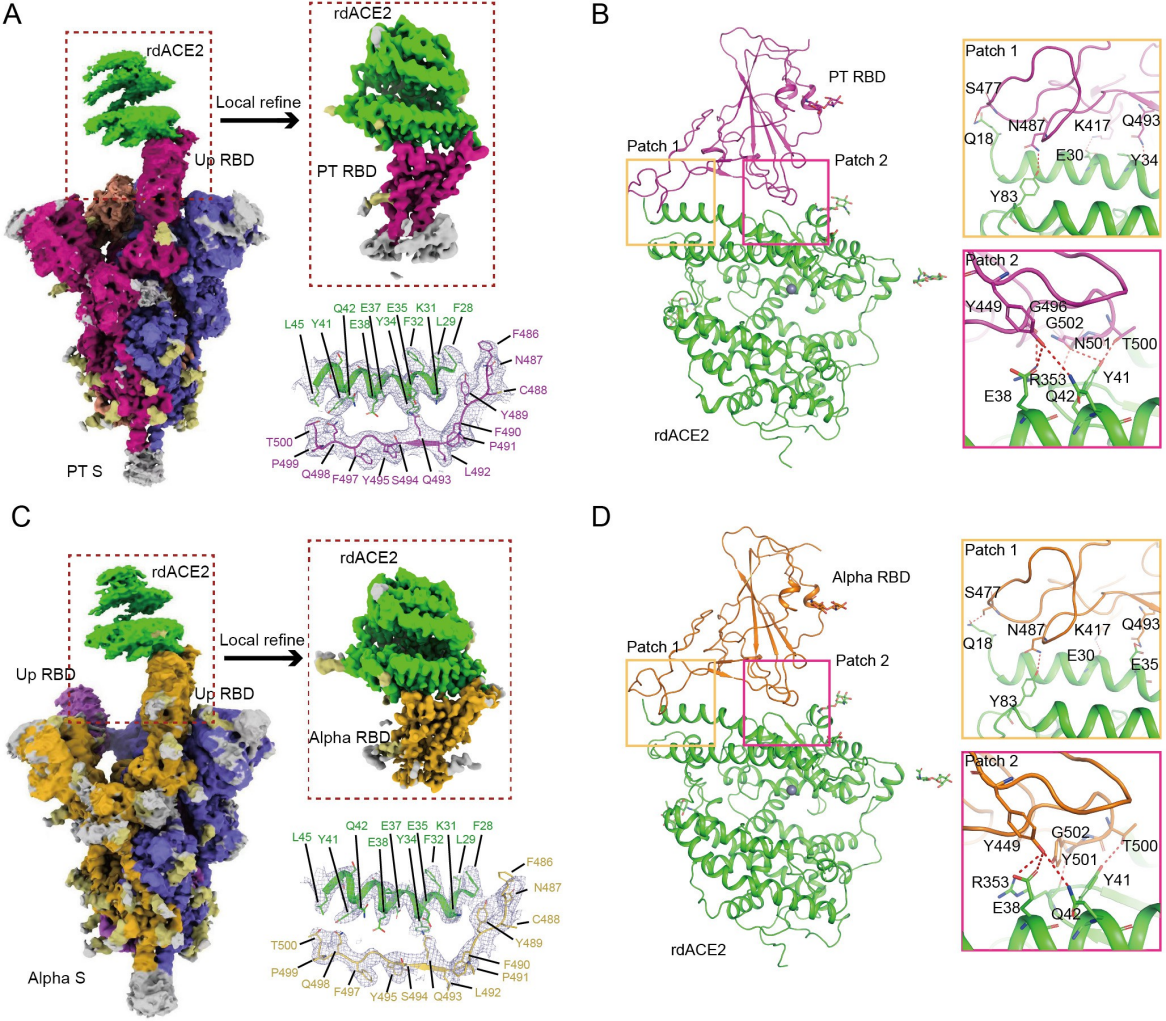
The structures of SARS-CoV-2 PT and Alpha S protein in complex with rdACE2. Cryo-EM density maps of the PT (A) and Alpha (C) S proteins bound to rdACE2 are depicted, with local refinement conducted on the binding interface of the S protein and rdACE2. The density maps illustrating the binding interface are presented as mesh, while the overall structures of rdACE2/PT RBD (B) and rdACE2/Alpha RBD (D) are represented as cartoons. Residues involved in the hydrogen bond networks of patch 1 and patch 2 are highlighted as sticks.

**Fig 3:**
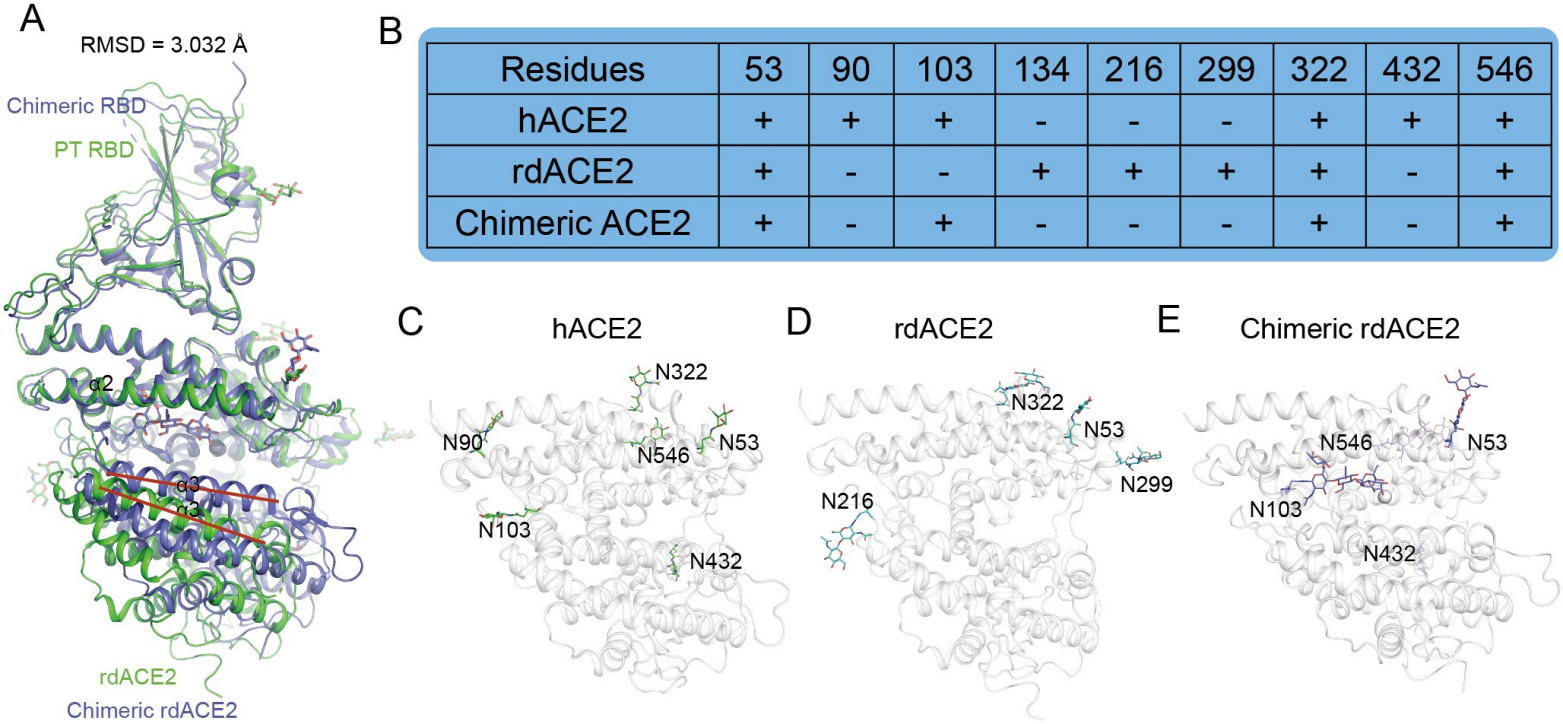
Differential N-glycosylation between hACE2 and rdACE2. (A) Superimposition of PT RBD/rdACE2 structure and chimeric RBD/chimeric rdACE2 strcture (PDB:8VQR). (B) Statistical analysis of N-glycosylations in hACE2 and rdACE2. (C-E) N-glycosylation modifications observed in the structures of hACE2, rdACE2, and chimeric rdACE2, respectively.

The cryo-EM structures of rdACE2 bound to SARS-CoV-2 PT or Alpha S proteins were determined at 2.52 Å and 2.86 Å, respectively **(S4 and S5 Figs and S1 Table)**. In the PT S/rdACE2 complex, one RBD of PT S was observed to interact with the peptidase domain (PD) of rdACE2, while the other two RBDs were in a downward conformation **(Fig 2A)**. Similarly, in the Alpha S/rdACE2 complex, two RBDs were present, but only one up-RBD was observed to bind to rdACE2 **(Fig 2C)**. To obtain a high-resolution view of the RBD/ACE2 interface, local refinement was performed, resulting in the determination of the PT RBD/rdACE2 and Alpha S RBD/rdACE2 structures at 2.64 Å and 3.16 Å, respectively **(S1 Table)**. The density of the interacting residues was clearly observed **(Figs 2A, 2C, S4 and S5)**. The overall structure of the PT RBD/rdACE2 complex exhibits significant divergence from that of the chimeric SARS-CoV-2 RBD/chimeric rdACE2 complex (PDB: 8VQR), with a root mean square deviation (RMSD) of 3.032 Å for all the Cα atoms **(Fig 3A).** Importantly, the chimeric rdACE2 is in a closed state, whereas both the crystal structure of hACE2 and the cryo-EM structure of rdACE2 determined by us are in an open state **(Fig 3A)** [20].

In the PD domain of hACE2, there are six N-linked glycosylation sites (N53, N90, N103, N322, N432 and N546). However, in the PD domain of rdACE2, N90, N103 and N432 are not part of the N-linked glycosylation motif sites. Instead, there are four additional N-linked glycosylation motifs in the rdACE2, including N134, N216 and N299 **(Fig 3B–3D)**. Six N-linked glycosylation motifs are situated in the PD of rdACE2, with four of them observed in the structure, except for N134 and N546 **(Fig 3C)**. Our previous study discovered that N-linked glycosylation of N90 in hACE2 forms an H-bond with T415 of the BA.2 RBD [39]. However, in rdACE2, this N-linked glycosylation motif is disrupted due to the N90D substitution **(S1 Fig)**. In the chimeric rdACE2, the N-linked glycosylation sites are the same as those in hACE2, except for residue 90, which does not have an N-linked glycosylation motif **(Fig 3D)**. Notably, in the chimeric rdACE2, the glycosylation at N103 is inserted into the gap between α2 and α4, whereas in hACE2, the glycosylation at N103 extends outward **(Fig 3C and 3E)**.

As previously reported, the interacting residues of the RBD can be divided into two patches [28]. In patch 1 of both PT and Alpha RBD **(Fig 2B and 2D and S2 Table)**, S477 and N487 form hydrogen bonds (H-bonds) with Q18 and Y83, respectively. K417 of RBD forms a salt bridge with E30 of rdACE2. In PT RBD, Q493 in patch 1 binds to Y34 of the rdACE2, whereas in Alpha RBD, Q493 forms an H-bond contact with E35. Moving to patch 2, T500 and N501 of both PT and Alpha RBD form H-bonds with Y41 of rdACE2, while G502 of these two RBDs forms an H-bond with R353 of rdACE2 **(Fig 2B and 2D and S2 Table)**. Furthermore, in PT RBD, Y449 forms an H-bond contact with E38 and Q42 of rdACE2 **(Fig 2B and S2 Table)**. Conversely, in Alpha RBD, Y449 forms an additional H-bond with R353 of rdACE2. Additionally, in PT RBD, G496 binds to E38 of rdACE2 via an H-bond **(Fig 2D and S2 Table)**.

### Distinct binding sites between hACE2 and rdACE2

**Fig 4:**
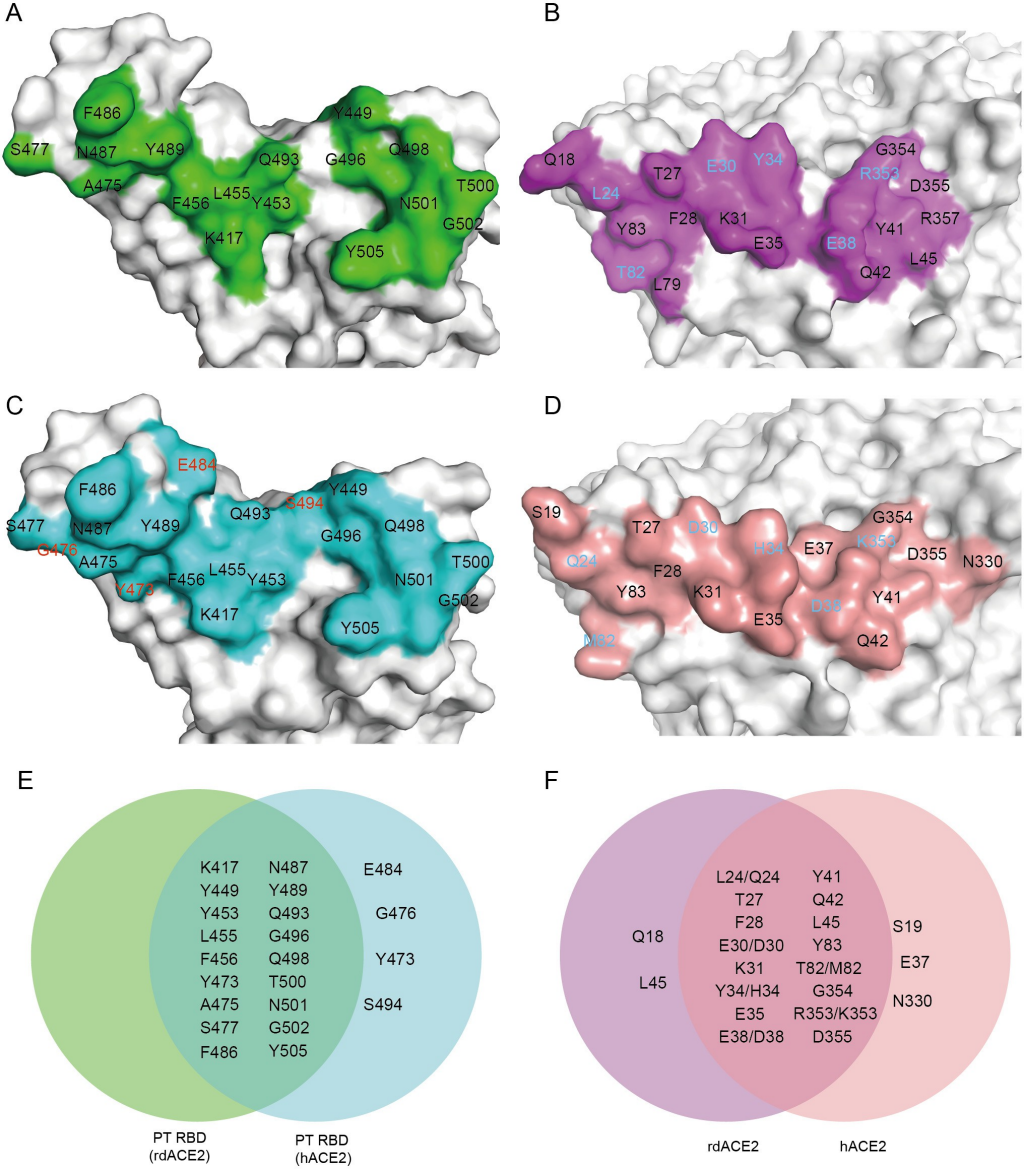
Comparison of the binding interfaces between PT RBDs with hACE2 or rdACE2. (A and B) The binding interfaces of PT RBD (A) and rdACE2 (B) in the rdACE2/PT RBD complex structure. (C and D) The binding interfaces of PT RBD (C) and hACE2 (D) in the hACE2/PT RBD complex structure. (E and F) Venn diagrams highlighting key residues at the binding interface. The red residues indicate those that interact with hACE2 but not with rdACE2. The cyan residues represent the distinct residues involved in PT RBD binding in both hACE2 and rdACE2.

We conducted a comparison of the binding interfaces of hACE2 (PDB: 7SXY) and rdACE2 to PT RBD. Through this structural analysis, we observed that the binding interface of PT RBD/hACE2 is larger than that of PT RBD/rdACE2 **(Fig 4A–4D)**. Specifically, residues Y473, G476, E484 and S494 of PT RBD were found to be involved in binding to hACE2 but not to rdACE2 **(Fig 4E)**.

Furthermore, we identified six distinct binding residues on the ACE2 interface that differ between rdACE2 and hACE2: L24 (rdACE2)/Q24 (hACE2), E30/D30, Y34/H34, E38/D38, T82M and R353/K353 **(Fig 4F)**. Additionally, residues Q18 and L45 of rdACE2 were found to participate in binding to PT RBD, whereas the corresponding residues of hACE2 do not contribute to binding **(Fig 4F)**. Conversely, residues S19, E37, and N330 of hACE2 interact with PT RBD, whereas rdACE2 lacks these interactions **(Fig 4F)**.

### The key residues for rdACE2 bound to SARS-CoV-2 RBD

To elucidate the molecular basis for the decreased affinity of rdACE2 for SARS-CoV-2 sub-variants, we constructed five rdACE2 mutants (L24Q, Y34H, E38D, T82M and R353K), substituting the corresponding residues with those of hACE2. Additionally, since N90-linked glycan of hACE2 has been observed to directly bind to BA.2, BA.2.86, and JN.1 RBDs, we created a rdACE2 D90N mutant.

**Fig 5:**
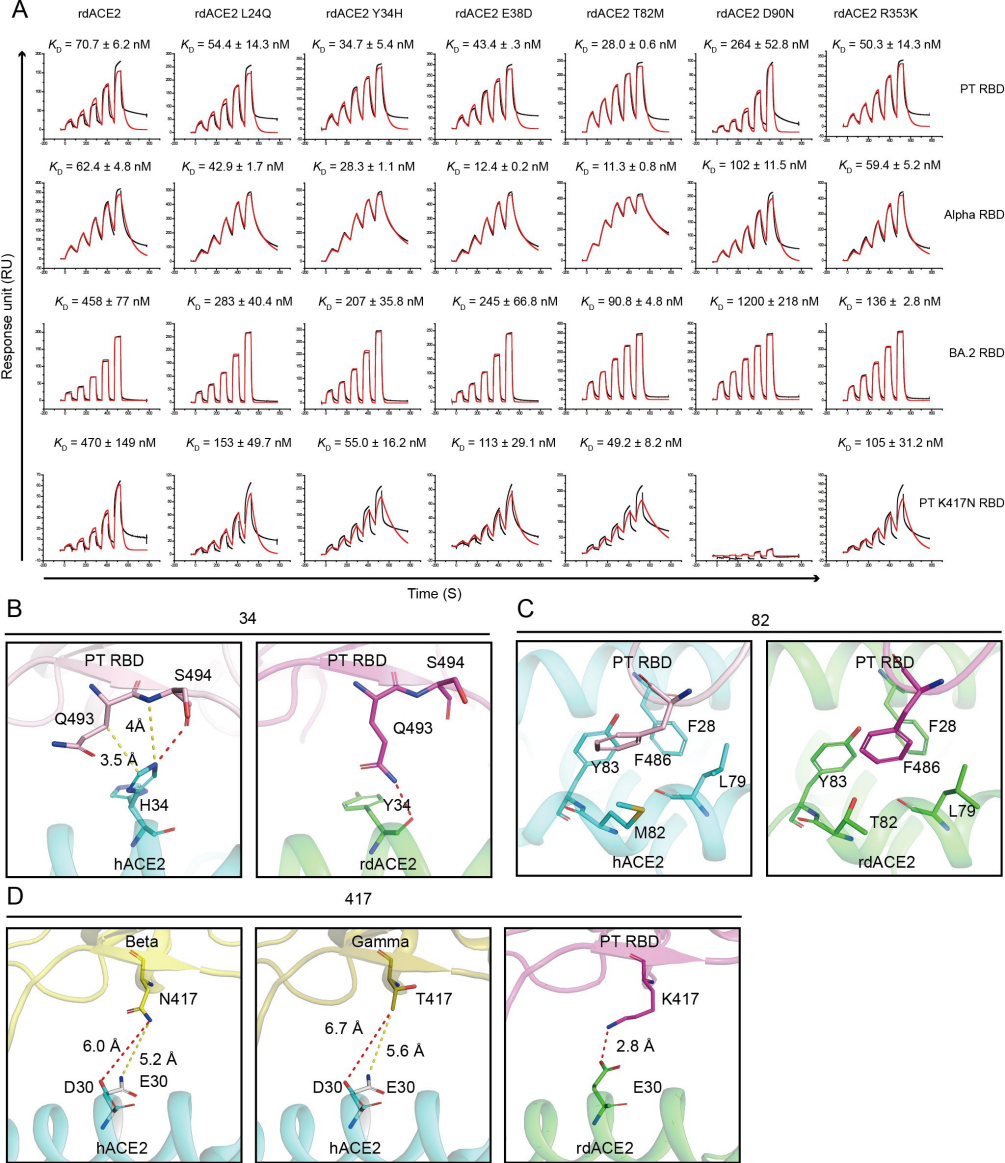
The critical residues involved in the binding of rdACE2 to SARS-CoV-2 RBDs. (A) Binding affinities of PT, Alpha, BA.2 and PT K417N RBDs to the different mutants of rdACE2. Raw curves are represented by black lines, and fitted curves are represented by red lines. (B-C) Structural comparison highlighting the designated key ACE2 residues (labeled above) that influence RBD binding, with these residues depicted as sticks. (D) Varying distances of K417/N417/T417 in the RBD to D30 (hACE2) or E30 (rdACE2).

We then tested the binding affinities of these mutants to the PT, Alpha and BA.2 RBDs, using hACE2 as a control **(S6 Fig)**. Our results showed that the Y34H and T82M mutants of rdACE2 exhibited more than a 2-fold increase in binding affinity compared to wild-type rdACE2 across all tested RBDs **(Fig 5A)**. Moreover, we found that the D90N mutant of rdACE2 decreased binding affinity for all tested RBDs **(Fig 5A)**.

When comparing the Beta and Gamma RBDs with the Alpha RBD, Beta and Gamma variants possess two substitutions, K417N/T and E484K. Although the binding of E484 to hACE2 is weak, K417 forms an H-bond with E30 of hACE2. Therefore, we also tested the binding affinities of the PT RBD K417N variant to wild-type and mutant rdACE2 proteins. The binding affinity of PT RBD K417N to wild-type rdACE2 decreased by more than 6-fold than PT RBD, but it only decreased by less than 2-fold when binding to the rdACE2 Y34H and T82M mutants **(Fig 5A)**.

From the structural analysis, we observed that in hACE2, H34 has two conformations, forming an H-bond with RBD S494 and engaging in Van der Waals’ force (vdw) interaction with RBD Q493 **(Fig 5B)**. Additionally, it can also form H-bonds with Q493 of RBDs from some SARS-CoV-2 sub-variants [38–41]. In rdACE2, the main chain of Y34 forms an H-bond with Q493 of RBD, but is far away from S494 of RBD **(Fig 5B)**. Furthermore, in hACE2, F28, L79, M82 and Y83 create a hydrophobic environment; however, when M82 of ACE2 is substituted by T82, this hydrophobic environment is disrupted, leading to decreased binding affinity **(Fig 5C)**. Moreover, RBD K417 forms an H-bond with E30 of rdACE2, but when it is mutated to RBD N417, the side chain becomes shorter and loses the H-bond contact **(Fig 5D)**.

## Discussion

The evolution of SARS-CoV-2 is constrained by the trade-off between enhancing immune evasion and maintaining an optimal binding affinity to its host receptor [38,42]. Previous studies have shown that the affinities of SARS-CoV-2 and its variants to hACE2 are limited within a certain range [38]. Additionally, it has been observed that SARS-CoV-2 can naturally infect white-tailed deer, mink, hamster and hippopotamuses. Of particular concern is the broad spread of SARS-CoV-2 among white-tailed deer, hamster and mink, leading to the emergence of new variants. Similar to hACE2, the binding of SARS-CoV-2 and most of its variants to ACE2 receptors from white-tailed deer, mink and hamsters is also limited within a certain range (within two orders of magnitude in nanomolar) [23,25,27].

In this study, we investigated the binding affinities of RBDs from various SARS-CoV-2 variants to rdACE2 receptor. We found that these affinities were generally lower compared to the PT RBD, falling into the range of three digital numbers. Notably, animal infection assays revealed that raccoon dogs typically exhibit mild clinical signs when infected with the PT virus. The decreased binding affinities observed in SARS-CoV-2 variants may indicate a reduced ability to infect raccoon dogs effectively.

From our structural analysis, we discovered that the rdACE2 mutants Y34H and T82M significantly enhance the binding affinity to the PT, Alpha, and BA.2 RBDs. More notably, the K417N mutation in the PT RBD substantially diminishes its binding to rdACE2. However, when the rdACE2 undergoes mutations from Y34 to H34 or from T82 to M82, these two mutants exhibit comparable binding affinities to the PT RBD K417N as seen in the original rdACE2/PT RBD interaction. This finding indicates that these two residues play a pivotal role in maintaining the binding affinity of ACE2 to different SARS-CoV-2 sub-variants.

Although structures determined by cryo-EM and those determined by crystallography may differ in their interaction details [40], the substantial differences in ACE2 conformation could be related to the protein constructs used. Additionally, the N103 glycosylation was not observed to insert into the gap between α2 and α4 in previously solved structures [20,28,39,40,43], which may be attributed to conformational effects caused by the chimeric constructs.

Some scientists raised the possibility that raccoon dogs could serve as natural hosts for SARS-CoV-2 [33, 34]. However, this finding alone falls short of conclusively establishing raccoon dogs as natural hosts. To date, while 45 species have been documented as naturally infected with SARS-CoV-2, there has been no confirmation of raccoon dogs being naturally infected with or carrying the virus (https://www.fao.org/animal-health/situation-updates/sars-cov-2-in-animals/en). Furthermore, investigations during the early stages of the COVID-19 epidemic failed to detect SARS-CoV-2 in raccoon dogs [35]. Thus, there is currently no direct evidence supporting that raccoon dogs are the natural hosts of SARS-CoV-2.

In summary, our data suggests that the binding affinities of rdACE2 to most of the SARS-CoV-2 sub-variants were reduced compared to PT. Additionally, structural analysis identified several key residues crucial for receptor binding. Our result indicated that the susceptibility of raccoon dog is lower than that of some animals with widespread transmission of SARS-CoV-2, such as white-tailed deer and hamster.

## Materials and methods

### Gene synthesis

The PD of the rdACE2 protein (UniProt, B4XEP4) and its mutants with the original signal peptide at the amino-terminal, and a 6×His tag connected to the carboxy-terminal (C-terminal), was designed to generate the deoxyribonucleic acid (DNA) sequence by codon-optimization. Then the DNA sequence was synthesized before being fused to the expression vector pcDNA3.1 [43].

For the S proteins of the SARS-CoV-2 PT and the Alpha variant, the residues 1–1208 were reserved while the transmembrane regions of the C-terminals (residues 1209–1273) were replaced by a trimerization tag (YIPEAPRDGQAYVRKDGEWVLLSTFL) followed by a strep tag (SAWSHPQFEK) and a 6×His tag. There were a "GSG" linker in front of the trimerization tag and a single “G” added the end of this tag. There were no linkers between other tags. The sequence was internally modified with the “6P” mutations reported to improve stability [44]. The codon-optimized DNA sequences were synthesized before being fused to the expression vector pCAGGS.

The RBD (corresponding to residues 319–541 of the S protein) of each variant was fused with a signal peptide from interleukin-10 (IL-10) at the amino-terminal, and a 6×His tag at the carboxy-terminal. The codon-optimized DNA sequences were synthesized before being fused to the expression vector pCAGGS, as previously reported [26].

All the genes and subcloned-plasmids mentioned above were produced by GenScript (www.genscript.com).

### Protein expression and purification

HEK293F cells (Gibco, Cat# 11625–019) were cultured at 37°C, 5% CO_2_ in SMM 293-TII expression medium (Sino Biological, Cat# M293TII) to express soluble proteins. When the cell density reached 2×10^6^ cells/ml, each plasmid and its three times mass of polyethylenimine (PEI) were separately diluted within phosphate buffered saline (PBS) and mixed for 20 minutes before being added to 293F cells, 100 μg plasmid was transfected into 100 ml cells. Five days later, the supernatants were centrifuged and filtered to remove the cell debris, then the His-tagged proteins were captured by the His columns (Cytiva, Cat# 17524802). All purification steps were carried out in a PBS-based buffer. Specifically, the irrelevant host proteins were washed off with the PBS solution containing 20 mM imidazole, while the target proteins were washed off and collected with the PBS solution containing 300 mM imidazole. The purified proteins were further purified by size exclusion chromatography using different types of molecular sieves, with the HiLoad 16/600 Superdex 200 pg (Cytiva, Cat# 28989335) for S protein and the Superose 6 Increase 10/300 GL (Cytiva, Cat# 29091596) for RBD and ACE2 proteins. The protein samples corresponding to the UV absorption peaks were collected and identified by SDS-PAGE (sodium dodecyl sulfate -polyacrylamide gel electrophoresis).

### SPR assays

All affinity assays were conducted in PBST (0.005% Tween-20 in PBS) and measured by a BIAcore 8K instrument (GE Healthcare) at 25°C. Before the measurements, the rdACE2 protein was immobilized on the CM5 sensor chip (Cytiva, Cat# 29149603) with amine coupling reaction, the target immobilization level 5000 response units (RU), and the actual immobilization levels were 1300–6700 RU. In the preliminary experiment, each RBD variant was diluted to concentrations ranging from 200 nM to 12.5 nM, and a single-cycle experiment was performed. To ensure that the objective values fall within the fitting range, the concentration ranges of the RBDs were adjusted as shown in **S3–S7 Tables**.

The *K*_D_ values from SPR experiments were obtained using BIAcore 8K evaluation software (GE Healthcare), with a 1:1 Langmuir binding model. Values of single-cycle analysis are the mean ± standard deviation (SD) of three independent experiments.

### Pseudovirus infection assay

BHK-21 cells were transfected with the pEGFP-N1-rdACE2s plasmids. After 24 h, the EGFP-positive cells were sorted, reseeded in 96-well plates at 2 × 10^4^ cells/well, and cultured for another 24 h. The pseudovirus particles of SARS-CoV-2 PT and its variants were diluted to the same amount, according to the RT-PCR results. Then, 100 μL of each pseudovirus was added to the sorted eGFP-positive cells. At 15 h post-transfection, imaging and analysis of fluorescent cells were performed using a CQ1 confocal image cytometer (Yokogawa, Japan). Each group contained three replicates.

### Cryo-sample preparation

Before cryo-EM sampling, all proteins were dialyzed from PBS to buffer A (20mM Tris, 150 mM NaCl, pH 8.0), and adjusted to 2 mg/ml. Then 5 μl rdACE2 was added into the equal volume of spike proteins, respectively. The mixtures were placed on ice for 1.5 h. At the same time, the 300 mash gold grids with 2 μm/2 μm C-flat films (Quantifoil) were glow-discharged in 15 mA electric current for 30 s using an easiGlow machine (PELCO). After being applied with 4μl protein solution, each grid was frozen in liquid ethane using a Vitrobot device (ThermoFisher Scientific), in which 4°C temperature, 100% humidity, 7 s blot time and −5 blot force was settled for all the samples. The grids were accepted into plastic boxes and stored in liquid nitrogen before use.

### Cryo-EM data collection

The grids were packed and loaded into a 300 kV Titan Krios transmission electron microscope equipped with Gatan K3 camera and matched with the EPU software (ThermoFisher Scientific). All the movies were collected at ×105,000 magnification with the pixel size of 0.69 Å over a defocus range of −1.0 μm to −2.0 μm in super-resolution counting mode with a total dose of 60 e^-^/Å^2^. Each movie contains 32 frames.

### Cryo-EM data processing

All the movies were processed on the cryoSPARC platform [45]. Generally, by Motion Correction [46] and CTF (contrast transfer function) estimation [47], each micrograph was generated from a movie, accordingly. The diameter ranges from 80 to 500 Å was set to picked out and extracted the particles, after 5–9 rounds of interactive 2D classification, serval particle stacks were selected for Ab-initial Reconstruction to generated 4~8 volumes, followed by the Heterogeneous Refinement [48,49]. And the best volumes were chosen for Non-uniform Refinement, CTF Refinement and DeepEMhancer sharpening [50]. The resolution was determined by the FCS cut-off at 0.143 [51]. To conduct the local refinements, partial maps of ACE2/RBD complexes were extracted from the overall maps utilizing the volume eraser tool in ChimeraX [52]. Subsequently, these partial maps were imported into Cryosparc to generate masks with a dilution radius of "6", a soft padding width of "3", and an appropriate threshold that minimally includes homologous structures. Each local map was produced through two iterations of localized refinements.

For the data set of PT RBD/rdACE2 complex, 17,940 micrographs were generated form the same number of movies, and 2,620,451 particles were extracted from the micrographs. After 2D-classfication, 1,452,120 particles were chosen for Ab-initial reconstruction, in which 309,215 particles were sorted out for the refinement of the final volume, and the resolution was calculated to be 2.52 Å. The local refinement was performed to generate the RBD/ACE2-focused map with the resolution of 2.64 Å (**S2 Fig**).

For the data set of Alpha RBD/rdACE2 complex, 6,307 micrographs were generated form the same number of movies, and 1,299,569 particles were extracted from the micrographs. After 2D-classfication, 396,183 particles were chosen for 3D reconstruction, in which 306,176 particles were sorted out for the refinement of the final volume, and the resolution was calculated to be 2.86 Å. The local refinement was performed to generate the RBD/ACE2-focused map with the resolution of 3.16 Å (**S3 Fig**).

### Structural model building and refinement

The PT RBD/ACE2 structure labeled 6LZG was used as the initial models. After molecular replacement, the structures were fitted to the local refined maps using ChimeraX and COOT [53], the main chains and side chains were adjusted manually. Then the maps together with the structures were processed by real-space refinement from PHINEX [54]. The structural figures were presented by PyMOL (https://pymol.org/).

Detailed information for the cryo-EM data set is available in **S1 Table**.

## Supporting information

S1 Fig Structure-based sequence alignment of ACE2 orthologs. Coils indicate α helices, black arrows indicate β strands and TT indicates β-turn. Conserved residues are highlighted in red. Sequence alignment is generated with Clustal X and ESPript 3.0.cc.
(PNG)

S2 Fig Residues mutated in RBD of SARS-CoV-2 VOCs were summarized. Residues that differ from the PT RBD are highlighted in different colors to indicate their variations.
(PNG)

S3 Fig The purity of the immobilized ligands was assessed using sodium dodecyl sulfate-polyacrylamide gel electrophoresis (SDS-PAGE). (TIF)

S4 Fig Cryo-EM data processing of the SARS-CoV-2 prototype RBD/rdACE2 complex. (A) A representative electron micrograph. (B) 2D classes selected for reconstruction. (C) Main steps of image processing. (D) Angular distribution of the particles. (E) Global and (F) local resolution estimation of the final volumes. (G) Global and (H) local resolution distribution of the cryo-EM maps, where blue represents for high-resolution areas, and red represents for low-resolution areas.
(PNG)

S5 Fig Cryo-EM data processing of the SARS-CoV-2 Alpha RBD/rdACE2 complex. (A) A representative electron micrograph. (B) 2D classes selected for reconstruction. (C) Main steps of image processing. (D) Angular distribution of the particles. (E) Global and (F) local resolution estimation of the final volumes. (G) Global and (H) local resolution distribution of the cryo-EM maps, where blue represents for high-resolution areas, and red represents for low-resolution areas.
(PNG)

S6 Fig The binding affinities of hACE2 to SARS-CoV-2 RBDs of PT, K417N, Alpha and BA.2. Raw curves are represented by black lines, and fitted curves are represented by red lines.
(TIF)

S1 Table Cryo-EM data collection, refinement, and validation statistics. (DOCX)

S2 Table The amino acid residues of SARS-CoV-2 PT and Alpha variant RBDs interact with rdACE2. (DOCX)

S3 Table The immobilization and concentrations statistics of SPR assay to test the binding affinities between rdACE2 and RBD. (DOCX)

S4 Table The immobilization and concentrations statistics of SPR assay to test the binding affinities between ACE2 and PT RBD. (DOCX)

S5 Table The immobilization and concentrations statistics of SPR assay to test the binding affinities between ACE2 and Alpha RBD. (DOCX)

S6 Table The immobilization and concentrations statistics of SPR assay to test the binding affinities between ACE2 and BA.2 RBD. (DOCX)

S7 Table The immobilization and concentrations statistics of SPR assay to test the binding affinities between ACE2 and PT K417N RBD. (DOCX)
