# Supplementary figures and images for: Receptor binding and structural basis of raccoon dog ACE2 binding to SARS-CoV-2 prototype and its variants
Source: PLoS Pathog. 2024 Dec 5;20(12):e1012713. doi: 10.1371/journal.ppat.1012713 (PMC11620640; doi:10.1371/journal.ppat.1012713)

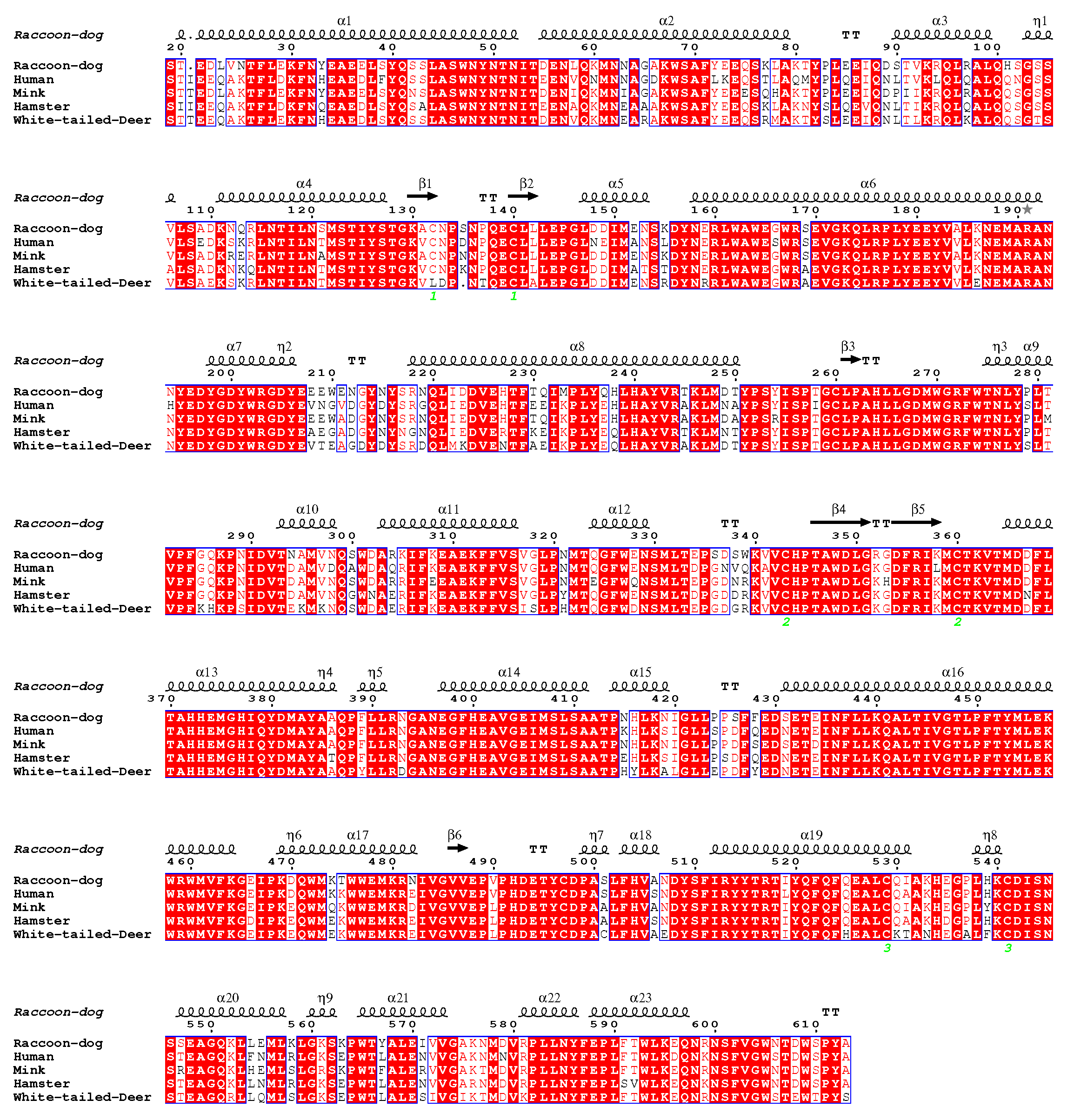

Supplement: S1 Fig — Coils indicate α helices, black arrows indicate β strands and TT indicates β-turn. Conserved residues are highlighted in red. Sequence alignment is generated with Clustal X and ESPript 3.0.cc. (PNG) [file ppat.1012713.s001.png]

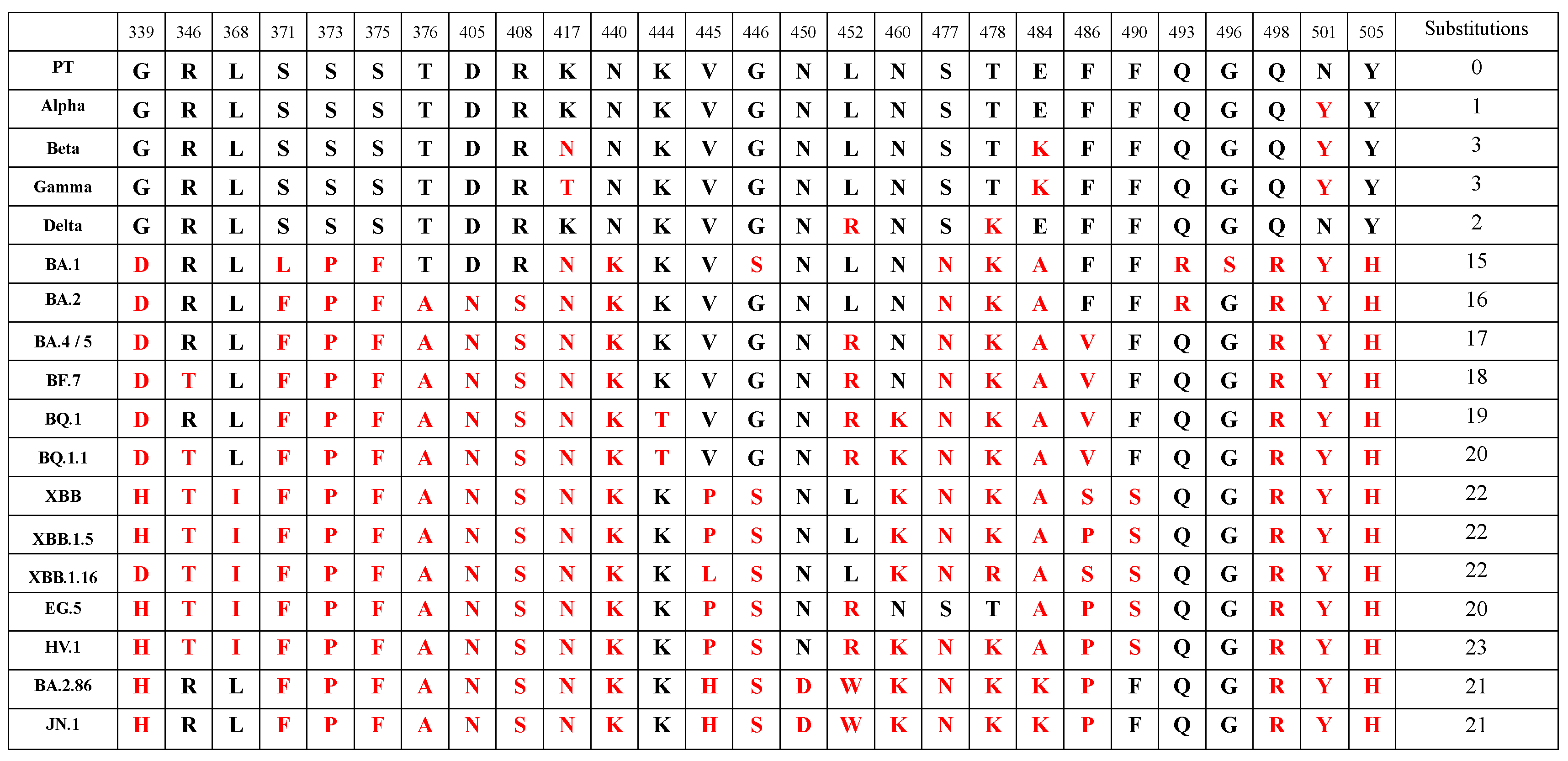

Supplement: S2 Fig — Residues that differ from the PT RBD are highlighted in different colors to indicate their variations. (PNG) [file ppat.1012713.s002.png]

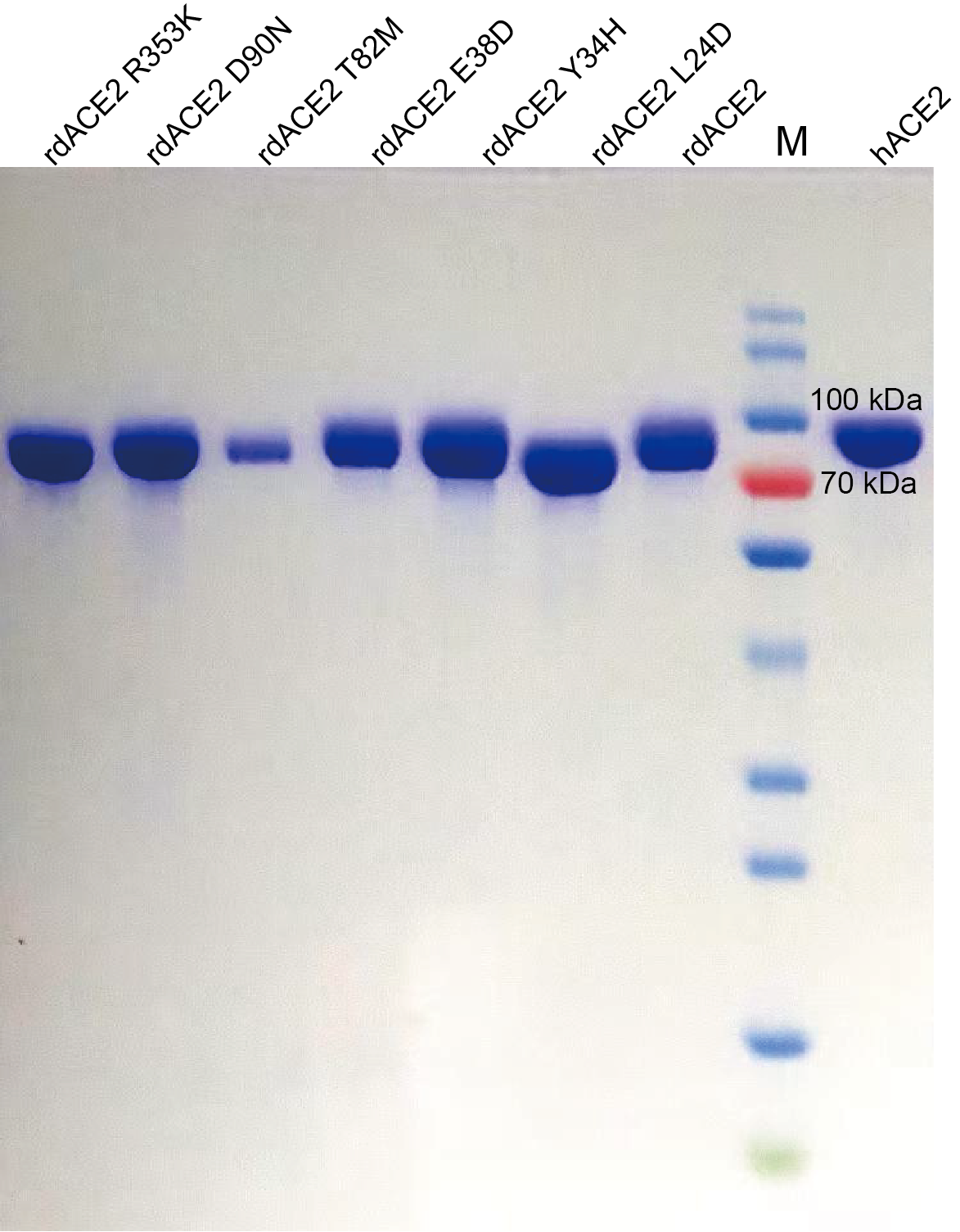

Supplement: S3 Fig — (TIF) [file ppat.1012713.s003.tif]

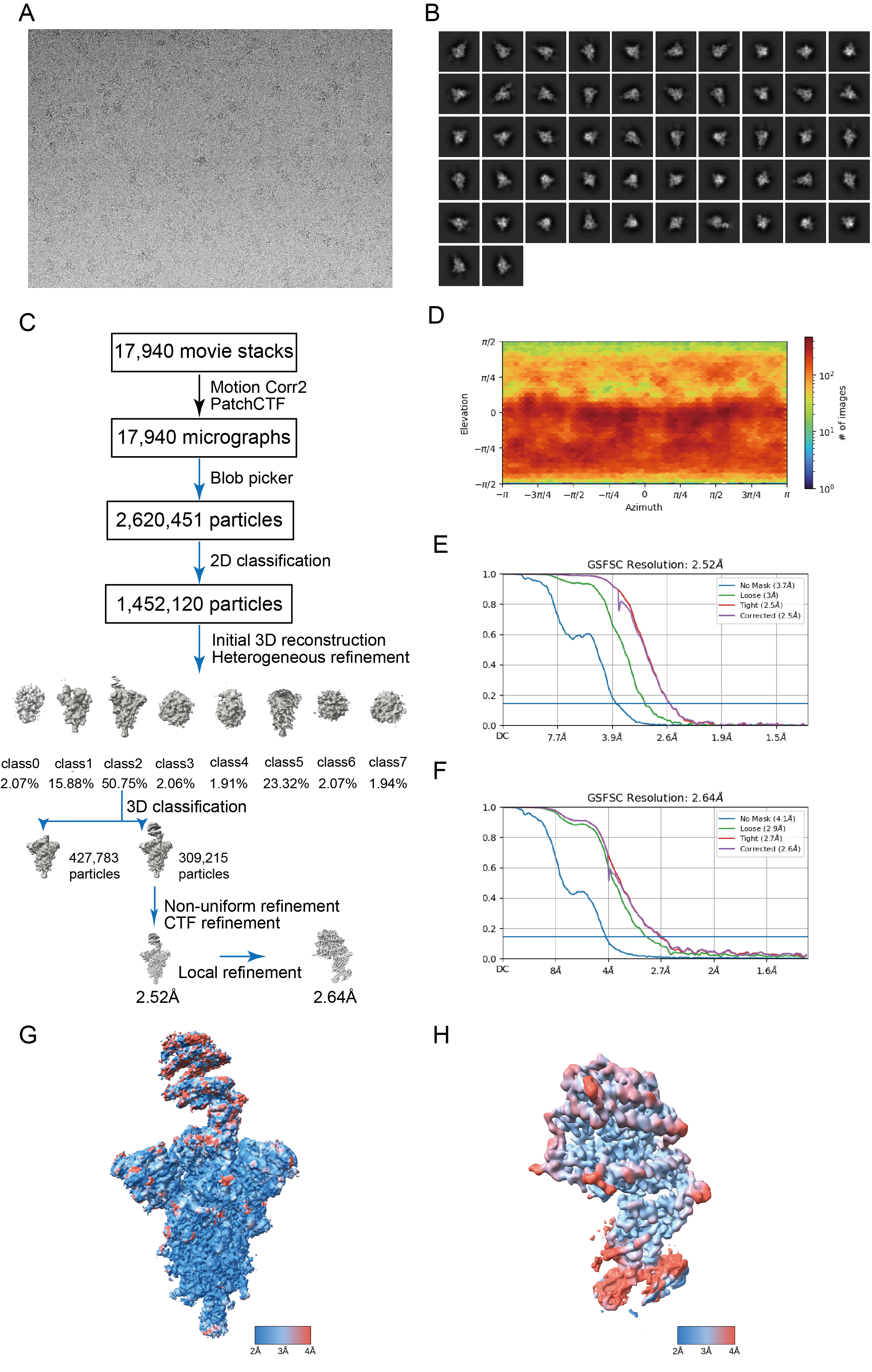

Supplement: S4 Fig — (A) A representative electron micrograph. (B) 2D classes selected for reconstruction. (C) Main steps of image processing. (D) Angular distribution of the particles. (E) Global and (F) local resolution estimation of the final volumes. (G) Global and (H) local resolution distribution of the cryo-EM maps, where blue represents for high-resolution areas, and red represents for low-resolution areas. (PNG) [file ppat.1012713.s004.png]

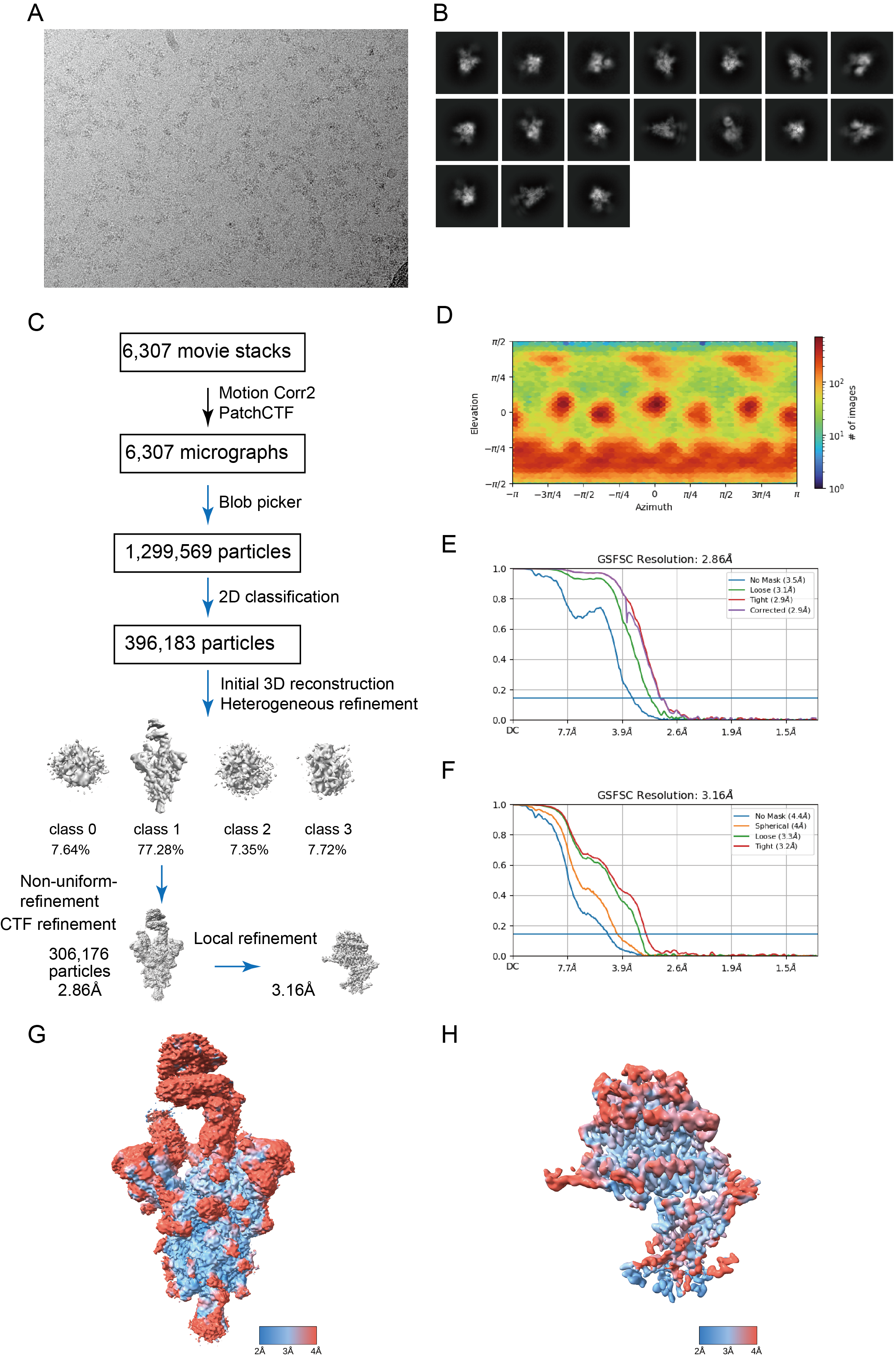

Supplement: S5 Fig — (A) A representative electron micrograph. (B) 2D classes selected for reconstruction. (C) Main steps of image processing. (D) Angular distribution of the particles. (E) Global and (F) local resolution estimation of the final volumes. (G) Global and (H) local resolution distribution of the cryo-EM maps, where blue represents for high-resolution areas, and red represents for low-resolution areas. (PNG) [file ppat.1012713.s005.png]

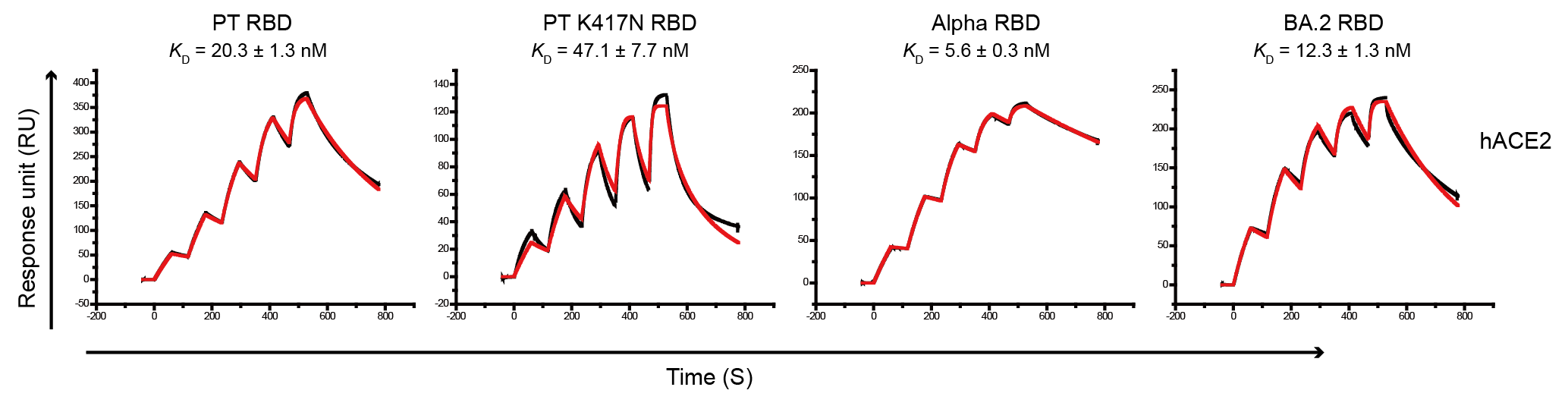

Supplement: S6 Fig — Raw curves are represented by black lines, and fitted curves are represented by red lines. (TIF) [file ppat.1012713.s006.tif]
