# Supplementary material for: Receptor binding and structural basis of raccoon dog ACE2 binding to SARS-CoV-2 prototype and its variants
Source: PLoS Pathog. 2024 Dec 5;20(12):e1012713. doi: 10.1371/journal.ppat.1012713 (PMC11620640; doi:10.1371/journal.ppat.1012713)
Supplement: S1 Table — (DOCX) [file ppat.1012713.s007.docx]

Table S1 Cryo-EM data collection, refinement, and validation statistics

|  | PT RBD/rdACE2 complex | Alpha RBD/rdACE2 complex |
| --- | --- | --- |
| **Data collection and processing** |  |  |
| Voltage (kV) | 300 | 300 |
| Electron exposure (e^-^/Å^2^) | 60 | 60 |
| Defocus range (μm) | -1.0 to -2.0 | -1.0 to -2.0 |
| Pixel size (Å) | 0.69 | 0.69 |
| Number of frames collected | 32 | 32 |
| Micrographs Collected (no.) | 17,940 | 6,307 |
| Symmetry imposed | C1 | C1 |
| Final particles (no.) | 309,215 | 306,176 |
| Map resolution (Å) | 2.64 | 3.16 |
| FSC threshold | 0.143 | 0.143 |
| **Refinement** |  |  |
| Initial model used (PDB code) | 6LZG/7Y9Z | 6LZG/7Y9Z |
| Map sharpening methods | DeepEMhancer | DeepEMhancer |
| **Model composition** |  |  |
| Non-hydrogen atoms | 6504 | 6508 |
| Protein residues | 783 | 783 |
| Ligands | Zn:1, NAG:9 | Zn:1, NAG:9 |
| **R.m.s. deviations** |  |  |
| Bond lengths (Å) | 0.003 | 0.004 |
| Bond angles (°) | 0.586 | 0.980 |
| **Validation** |  |  |
| MolProbity Score | 1.41 | 1.50 |
| Clash Score | 3.79 | 4.18 |
| Poor rotamers (%) | 0.43 | 0.72 |
| **Ramachandran plot** |  |  |
| Favored (%) | 96.40 | 95.62 |
| Allowed (%) | 3.47 | 4.12 |
| Disallowed (%) | 0.13 | 0.26 |
