# Supplementary material for: Receptor binding and structural basis of raccoon dog ACE2 binding to SARS-CoV-2 prototype and its variants
Source: PLoS Pathog. 2024 Dec 5;20(12):e1012713. doi: 10.1371/journal.ppat.1012713 (PMC11620640; doi:10.1371/journal.ppat.1012713)
Supplement: S2 Table — (DOCX) [file ppat.1012713.s008.docx]

**Table S2 The amino acid residues of SARS-CoV-2 PT and Alpha variant RBDs interact with rdACE2**

| **rdACE2** | **PT RBD** | **Alpha RBD** |
| --- | --- | --- |
| Q19 | S477 (12) | S477 (9) |
| L24 | A475 (3), N487 (5) | A475 (3), G476 (2), N487 (3) |
| T27 | F456 (5), Y489 (3) | F456 (4), A475 (1), Y489 (3) |
| F28 | Y489 (5) | Y489 (6) |
| E30 | K417 (6, **1**) | K417 (5, **1**) |
| K31 | F456 (2), Y489 (2) | Y489 (6) |
| Y34 | K417 (3), Y453 (4), L455 (1), Q493 (13, **1**) | Y453 (6), Q493 (8) |
| E35 | Q493 (7) | Q493 (11, **1**) |
| E38 | Y449 (11, **1**) | Y449 (11, **1**), G496 (1) |
| Y41 | Q498 (4), T500 (7, **1**), N501 (6) | Q498 (8), T500 (4, **1**), Y501 (19) |
| Q42 | Y449 (1, **1**) | Y449 (1, **1**) |
| L45 | Q498 (2) | Q498 (1) |
| L79 | F486 (5) | F486 (8) |
| T82 | F486 (5) | F486 (3) |
| Y83 | F486 (4), N487 (4, **1**) | F486 (6), N487 (6, **1**), Y489 (1) |
| N330 |  | T500 (3) |
| R353 | G496 (4, **1**), N501 (10), G502 (3, **1**), Y505 (20) | Y501 (17), G502 (3, **1**), Y505 (8) |
| G354 | G502 (6), Y505 (1) | G502 (6) |
| D355 | T500 (7) | T500 (5) |
| R357 | T500 (3) | T500 (3) |
| **Total** | 174, **8** | 182, **7** |

The numbers in parentheses of PT RBD and Alpha RBD residues represent the number of vdw contacts between the indicated residues of rdACE2 with PT RBD or Alpha RBD. The numbers with underline suggest numbers of potential H-bonds between the pairs of residues. wdw contact was analyzed at a cutoff of 4.5 Å and H-bonds at a cutoff of 3.5.
