# Supplementary material for: Receptor binding and structural basis of raccoon dog ACE2 binding to SARS-CoV-2 prototype and its variants
Source: PLoS Pathog. 2024 Dec 5;20(12):e1012713. doi: 10.1371/journal.ppat.1012713 (PMC11620640; doi:10.1371/journal.ppat.1012713)
Supplement: S3 Table — (DOCX) [file ppat.1012713.s009.docx]

**Table S3** The immobilization and concentrations statistics of SPR assay to test the binding affinities between rdACE2 and RBD

| **Ligand** | **Immobilization quantity (units)** | **Concentrations of RBD**  **(nM)** | ***k*_a_ (1/Ms)** | ***k*_d_ (1/s)** | ***K*_D_ (M)** | **Average *K*_D_ (M)** | **SD  (M)** |
| --- | --- | --- | --- | --- | --- | --- | --- |
| PT RBD | 1356.8 | 400, 200, 100, 50, 25 | 1.64*10^6^ | 9.85*10^-2^ | 6.01*10^-8^ | 8.74*10^-8^ | 2.38*10^-8^ |
|  |  |  | 2.95*10^6^ | 2.48*10^-1^ | 8.40*10^-8^ |  |  |
|  |  |  | 2.54*10^5^ | 3.00*10^-2^ | 1.18*10^-7^ |  |  |
| Alpha RBD | 1441.7 | 400, 200, 100, 50, 25 | 2.19*10^6^ | 1.24*10^-1^ | 5.64*10^-8^ | 7.67*10^-8^ | 1.47*10^-8^ |
|  |  |  | 8.69*10^5^ | 7.21*10^-2^ | 8.29*10^-8^ |  |  |
|  |  |  | 3.03*10^5^ | 2.75*10^-2^ | 9.08*10^-8^ |  |  |
| Beta RBD | 1326.9 | 400, 200, 100, 50, 25 | 1.01*10^5^ | 3.62*10^-2^ | 3.60*10^-7^ | 3.92*10^-7^ | 2.31*10^-8^ |
|  |  |  | 1.23*10^8^ | 4.90*10^1^ | 3.99*10^-7^ |  |  |
|  |  |  | 7.55*10^4^ | 3.13*10^-2^ | 4.15*10^-7^ |  |  |
| Gamma RBD | 1402.8 | 400, 200, 100, 50, 25 | 1.21*10^5^ | 2.40*10^-2^ | 1.99*10^-7^ | 2.08*10^-7^ | 2.54*10^-8^ |
|  |  |  | 1.13*10^5^ | 2.07*10^-2^ | 1.83*10^-7^ |  |  |
|  |  |  | 9.45*10^4^ | 2.30*10^-2^ | 2.43*10^-7^ |  |  |
| Delta RBD | 1397.1 | 400, 200, 100, 50, 25 | 3.35*10^5^ | 2.40*10^-2^ | 7.17*10^-8^ | 9.59*10^-8^ | 2.14*10^-8^ |
|  |  |  | 2.77*10^5^ | 2.55*10^-2^ | 9.21*10^-8^ |  |  |
|  |  |  | 2.33*10^5^ | 2.88*10^-2^ | 1.24*10^-7^ |  |  |
| BA.1 RBD | 1395.3 | 400, 200, 100, 50, 25 | 3.19*10^5^ | 9.85*10^-2^ | 3.09*10^-7^ | 4.29*10^-7^ | 9.30*10^-8^ |
|  |  |  | 2.50*10^5^ | 1.10*10^-1^ | 4.41*10^-7^ |  |  |
|  |  |  | 2.10*10^5^ | 1.13*10^-1^ | 5.36*10^-7^ |  |  |
| BA.2 RBD | 1420.9 | 400, 200, 100, 50, 25 | 1.94*10^5^ | 1.11*10^-1^ | 5.71*10^-7^ | 8.11*10^-7^ | 2.05*10^-7^ |
|  |  |  | 1.02*10^5^ | 8.08*10^-2^ | 7.92*10^-7^ |  |  |
|  |  |  | 2.41*10^5^ | 2.59*10^-1^ | 1.07*10^-6^ |  |  |
| BA.4/5 RBD | 1401.9 | 400, 200, 100, 50, 25 | 7.20*10^4^ | 4.13*10^-2^ | 5.74*10^-7^ | 5.40*10^-7^ | 7.86*10^-8^ |
|  |  |  | 5.61*10^4^ | 3.45*10^-2^ | 6.15*10^-7^ |  |  |
|  |  |  | 7.41*10^4^ | 3.20*10^-2^ | 4.31*10^-7^ |  |  |
| BQ.1 RBD | 1356.8 | 400, 200, 100, 50, 25 | 3.17*10^5^ | 7.52*10^-2^ | 2.37*10^-7^ | 3.11*10^-7^ | 1.12*10^-7^ |
|  |  |  | 2.69*10^5^ | 6.11*10^-2^ | 2.27*10^-7^ |  |  |
|  |  |  | 1.65*10^5^ | 7.73*10^-2^ | 4.69*10^-7^ |  |  |
| BQ.1.1 RBD | 1441.7 | 400, 200, 100, 50, 25 | 1.33*10^5^ | 3.77*10^-2^ | 2.84*10^-7^ | 3.21*10^-7^ | 3.61*10^-8^ |
|  |  |  | 1.15*10^5^ | 3.56*10^-2^ | 3.10*10^-7^ |  |  |
|  |  |  | 1.01*10^5^ | 3.73*10^-2^ | 3.70*10^-7^ |  |  |
| BF.7 RBD | 1326.9 | 400, 200, 100, 50, 25 | 4.63*10^4^ | 2.97*10^-2^ | 6.42*10^-7^ | 5.97*10^-7^ | 1.74*10^-7^ |
|  |  |  | 7.08*10^4^ | 2.59*10^-2^ | 3.66*10^-7^ |  |  |
|  |  |  | 3.64*10^4^ | 2.86*10^-2^ | 7.84*10^-7^ |  |  |
| XBB RBD | 1402.8 | 400, 200, 100, 50, 25 | 2.11*10^5^ | 1.67*10^-1^ | 7.95*10^-7^ | 7.20*10^-7^ | 5.66*10^-8^ |
|  |  |  | 2.00*10^5^ | 1.41*10^-1^ | 7.07*10^-7^ |  |  |
|  |  |  | 2.09*10^5^ | 1.37*10^-1^ | 6.58*10^-7^ |  |  |
| XBB.1.5 RBD | 1397.1 | 400, 200, 100, 50, 25 | 9.97*10^4^ | 4.61*10^-2^ | 4.62*10^-7^ | 4.33*10^-7^ | 2.40*10^-8^ |
|  |  |  | 8.66*10^4^ | 3.49*10^-2^ | 4.03*10^-7^ |  |  |
|  |  |  | 7.70*10^4^ | 3.34*10^-2^ | 4.34*10^-7^ |  |  |
| XBB.1.16 RBD | 1395.3 | 400, 200, 100, 50, 25 | 1.10*10^5^ | 3.13*10^-2^ | 2.85*10^-7^ | 3.02*10^-7^ | 2.49*10^-8^ |
|  |  |  | 9.78*10^4^ | 2.79*10^-2^ | 2.85*10^-7^ |  |  |
|  |  |  | 8.66*10^4^ | 2.92*10^-2^ | 3.37*10^-7^ |  |  |
| EG.5 RBD | 1420.9 | 400, 200, 100, 50, 25 | 1.01*10^5^ | 4.90*10^-2^ | 4.84*10^-7^ | 4.26*10^-7^ | 4.75*10^-8^ |
|  |  |  | 1.14*10^5^ | 4.18*10^-2^ | 3.68*10^-7^ |  |  |
|  |  |  | 1.01*10^5^ | 4.27*10^-2^ | 4.25*10^-7^ |  |  |
| HV.1 RBD | 1401.9 | 400, 200, 100, 50, 25 | 1.12*10^5^ | 5.10*10^-2^ | 4.55*10^-7^ | 4.79*10^-7^ | 3.71*10^-8^ |
|  |  |  | 1.04*10^5^ | 4.68*10^-2^ | 4.52*10^-7^ |  |  |
|  |  |  | 9.02*10^4^ | 4.80*10^-2^ | 5.32*10^-7^ |  |  |
| BA.2.86 RBD | 1356.8 | 400, 200, 100, 50, 25 | 5.27*10^4^ | 1.60*10^-2^ | 3.04*10^-7^ | 2.76*10^-7^ | 2.30*10^-8^ |
|  |  |  | 5.78*10^4^ | 1.59*10^-2^ | 2.75*10^-7^ |  |  |
|  |  |  | 6.28*10^4^ | 1.56*10^-2^ | 2.48*10^-7^ |  |  |
| JN.1 RBD | 1441.7 | 400, 200, 100, 50, 25 | 3.55*10^4^ | 1.77*10^-2^ | 5.00*10^-7^ | 4.20*10^-7^ | 6.26*10^-8^ |
|  |  |  | 5.03*10^4^ | 2.08*10^-2^ | 4.13*10^-7^ |  |  |
|  |  |  | 4.43*10^4^ | 1.54*10^-2^ | 3.48*10^-7^ |  |  |
