# Supplementary material for: Receptor binding and structural basis of raccoon dog ACE2 binding to SARS-CoV-2 prototype and its variants
Source: PLoS Pathog. 2024 Dec 5;20(12):e1012713. doi: 10.1371/journal.ppat.1012713 (PMC11620640; doi:10.1371/journal.ppat.1012713)
Supplement: S4 Table — (DOCX) [file ppat.1012713.s010.docx]

**Table S4** The immobilization and concentrations statistics of SPR assay to test the binding affinities between ACE2 and PT RBD

| **Ligand** | **Immobilization quantity (units)** | **Concentrations of RBD**  **(nM)** | ***k*_a_ (1/Ms)** | ***k*_d_ (1/s)** | ***K*_D_ (M)** | **Average *K*_D_ (M)** | **SD  (M)** |
| --- | --- | --- | --- | --- | --- | --- | --- |
| rdACE2 | 2513.7 | 200, 100, 50, 25, 12.5 | 4.69*10^5^ | 3.38*10^-2^ | 7.20*10^-8^ | 7.20*10^-8^ | 6.15*10^-9^ |
|  |  |  | 3.86*10^5^ | 2.99*10^-2^ | 7.74*10^-8^ |  |  |
|  |  |  | 7.02*10^5^ | 4.39*10^-2^ | 6.26*10^-8^ |  |  |
| rdACE2 L24Q | 2613.3 | 200, 100, 50, 25, 12.5 | 5.66*10^5^ | 3.60*10^-2^ | 6.35*10^-8^ | 5.44*10^-8^ | 1.43*10^-8^ |
|  |  |  | 5.87*10^5^ | 3.84*10^-2^ | 6.55*10^-8^ |  |  |
|  |  |  | 9.22*10^5^ | 3.15*10^-2^ | 3.41*10^-8^ |  |  |
| rdACE2 Y34H | 3222.0 | 200, 100, 50, 25, 12.5 | 6.81*10^5^ | 2.60*10^-2^ | 3.82*10^-8^ | 3.47*10^-8^ | 5.37*10^-9^ |
|  |  |  | 7.29*10^5^ | 2.83*10^-2^ | 3.89*10^-8^ |  |  |
|  |  |  | 8.09*10^5^ | 2.20*10^-2^ | 2.71*10^-8^ |  |  |
| rdACE2 E38D | 2897.8 | 200, 100, 50, 25, 12.5 | 6.49*10^5^ | 3.09*10^-2^ | 4.76*10^-8^ | 4.34*10^-8^ | 6.33*10^-9^ |
|  |  |  | 7.04*10^5^ | 3.39*10^-2^ | 4.82*10^-8^ |  |  |
|  |  |  | 7.72*10^5^ | 2.66*10^-2^ | 3.45*10^-8^ |  |  |
| rdACE2 T82M | 1766.3 | 200, 100, 50, 25, 12.5 | 8.88*10^5^ | 2.41*10^-2^ | 2.72*10^-8^ | 2.80*10^-8^ | 5.94*10^-10^ |
|  |  |  | 9.31*10^5^ | 2.61*10^-2^ | 2.81*10^-8^ |  |  |
|  |  |  | 8.08*10^5^ | 2.31*10^-2^ | 2.86*10^-8^ |  |  |
| rdACE2 D90N | 2691.4 | 200, 100, 50, 25, 12.5 | 4.35*10^5^ | 1.30*10^-1^ | 2.99*10^-7^ | 2.64*10^-7^ | 5.28*10^-8^ |
|  |  |  | 3.66*10^5^ | 6.93*10^-2^ | 1.89*10^-7^ |  |  |
|  |  |  | 3.25*10^5^ | 9.85*10^-2^ | 3.03*10^-7^ |  |  |
| rdACE2 R353K | 2524.7 | 200, 100, 50, 25, 12.5 | 5.42*10^5^ | 3.06*10^-2^ | 5.64*10^-8^ | 5.03*10^-8^ | 1.43*10^-9^ |
|  |  |  | 5.27*10^5^ | 3.37*10^-2^ | 6.40*10^-8^ |  |  |
|  |  |  | 7.55*10^5^ | 2.31*10^-2^ | 3.05*10^-8^ |  |  |
| hACE2 | 6626.4 | 400, 200, 100, 50, 25 | 2.10*10^5^ | 3.88*10^-2^ | 1.85*10^-8^ | 2.03*10^-8^ | 1.28*10^-9^ |
|  |  |  | 1.56*10^5^ | 3.36*10^-2^ | 2.16*10^-8^ |  |  |
|  |  |  | 1.60*10^5^ | 3.31*10^-2^ | 2.07*10^-8^ |  |  |
