# Supplementary material for: Receptor binding and structural basis of raccoon dog ACE2 binding to SARS-CoV-2 prototype and its variants
Source: PLoS Pathog. 2024 Dec 5;20(12):e1012713. doi: 10.1371/journal.ppat.1012713 (PMC11620640; doi:10.1371/journal.ppat.1012713)
Supplement: S5 Table — (DOCX) [file ppat.1012713.s011.docx]

**Table S5** The immobilization and concentrations statistics of SPR assay to test the binding affinities between ACE2 and Alpha RBD

| **Ligand** | **Immobilization quantity (units)** | **Concentrations of RBD**  **(nM)** | ***k*_a_ (1/Ms)** | ***k*_d_ (1/s)** | ***K*_D_ (M)** | **Average *K*_D_ (M)** | **SD  (M)** |
| --- | --- | --- | --- | --- | --- | --- | --- |
| rdACE2 | 2621.0 | 200, 100, 50, 25, 12.5 | 1.97*10^5^ | 1.35*10^-2^ | 6.87*10^-8^ | 6.24*10^-8^ | 4.84*10^-9^ |
|  |  |  | 1.37*10^7^ | 7.81*10^-1^ | 5.69*10^-8^ |  |  |
|  |  |  | 1.92*10^5^ | 1.19*10^-2^ | 6.18*10^-8^ |  |  |
| rdACE2 L24Q | 2738.0 | 200, 100, 50, 25, 12.5 | 7.80*10^5^ | 3.52*10^-2^ | 4.51*10^-8^ | 4.29*10^-8^ | 1.73*10^-9^ |
|  |  |  | 6.19*10^5^ | 2.64*10^-2^ | 4.26*10^-8^ |  |  |
|  |  |  | 6.58*10^5^ | 2.69*10^-2^ | 4.09*10^-8^ |  |  |
| rdACE2 Y34H | 3120.9 | 200, 100, 50, 25, 12.5 | 4.34*10^5^ | 1.28*10^-2^ | 2.96*10^-8^ | 2.83*10^-8^ | 1.13*10^-9^ |
|  |  |  | 3.81*10^5^ | 1.09*10^-2^ | 2.85*10^-8^ |  |  |
|  |  |  | 3.68*10^5^ | 9.89*10^-3^ | 2.69*10^-8^ |  |  |
| rdACE2 E38D | 2986.9 | 200, 100, 50, 25, 12.5 | 4.17*10^5^ | 5.26*10^-3^ | 1.26*10^-8^ | 1.24*10^-8^ | 1.55*10^-10^ |
|  |  |  | 3.78*10^5^ | 4.67*10^-3^ | 1.24*10^-8^ |  |  |
|  |  |  | 3.14*10^5^ | 3.85*10^-3^ | 1.22*10^-8^ |  |  |
| rdACE2 T82M | 1877.3 | 200, 100, 50, 25, 12.5 | 5.92*10^5^ | 7.35*10^-3^ | 1.24*10^-8^ | 1.13*10^-8^ | 8.22*10^-10^ |
|  |  |  | 6.27*10^5^ | 6.77*10^-3^ | 1.08*10^-8^ |  |  |
|  |  |  | 5.18*10^5^ | 5.48*10^-3^ | 1.06*10^-8^ |  |  |
| rdACE2 D90N | 2747.9 | 200, 100, 50, 25, 12.5 | 1.57*10^5^ | 1.85*10^-2^ | 1.18*10^-7^ | 1.02*10^-7^ | 1.15*10^-8^ |
|  |  |  | 1.68*10^5^ | 1.57*10^-2^ | 9.35*10^-8^ |  |  |
|  |  |  | 1.70*10^5^ | 1.60*10^-2^ | 9.41*10^-8^ |  |  |
| rdACE2 R353K | 2653.6 | 200, 100, 50, 25, 12.5 | 7.78*10^6^ | 4.10*10^-1^ | 5.27*10^-8^ | 5.94*10^-8^ | 5.19*10^-9^ |
|  |  |  | 1.17*10^7^ | 7.05*10^-1^ | 6.02*10^-8^ |  |  |
|  |  |  | 1.80*10^5^ | 1.18*10^-2^ | 6.53*10^-8^ |  |  |
| hACE2 | 6150.5 | 400, 200, 100, 50, 25 | 1.58*10^5^ | 8.21*10^-4^ | 5.21*10^-9^ | 5.58*10^-9^ | 2.92*10^-10^ |
|  |  |  | 1.66*10^5^ | 9.30*10^-4^ | 5.61*10^-9^ |  |  |
|  |  |  | 1.49*10^5^ | 8.85*10^-4^ | 5.92*10^-9^ |  |  |
