# Supplementary material for: Receptor binding and structural basis of raccoon dog ACE2 binding to SARS-CoV-2 prototype and its variants
Source: PLoS Pathog. 2024 Dec 5;20(12):e1012713. doi: 10.1371/journal.ppat.1012713 (PMC11620640; doi:10.1371/journal.ppat.1012713)
Supplement: S6 Table — (DOCX) [file ppat.1012713.s012.docx]

**Table S6** The immobilization and concentrations statistics of SPR assay to test the binding affinities between ACE2 and BA.2 RBD

| **Ligand** | **Immobilization quantity (units)** | **Concentrations of RBD**  **(nM)** | ***k*_a_ (1/Ms)** | ***k*_d_ (1/s)** | ***K*_D_ (M)** | **Average *K*_D_ (M)** | **SD  (M)** |
| --- | --- | --- | --- | --- | --- | --- | --- |
| rdACE2 | 2621.0 | 400, 200, 100, 50, 25 | 8.85*10^5^ | 3.09*10^-1^ | 3.50*10^-7^ | 4.58*10^-7^ | 7.70*10^-8^ |
|  |  |  | 5.61*10^5^ | 2.84*10^-1^ | 5.06*10^-7^ |  |  |
|  |  |  | 5.61*10^5^ | 2.92*10^-1^ | 5.20*10^-7^ |  |  |
| rdACE2 L24Q | 2738.0 | 400, 200, 100, 50, 25 | 8.75*10^5^ | 1.98*10^-1^ | 2.27*10^-7^ | 2.83*10^-7^ | 4.04*10^-8^ |
|  |  |  | 6.26*10^5^ | 1.92*10^-1^ | 3.06*10^-7^ |  |  |
|  |  |  | 6.17*10^5^ | 1.96*10^-1^ | 3.17*10^-7^ |  |  |
| rdACE2 Y34H | 3120.9 | 400, 200, 100, 50, 25 | 8.53*10^5^ | 1.35*10^-1^ | 1.58*10^-7^ | 2.07*10^-7^ | 3.58*10^-8^ |
|  |  |  | 5.98*10^5^ | 1.33*10^-1^ | 2.23*10^-7^ |  |  |
|  |  |  | 5.86*10^5^ | 1.41*10^-1^ | 2.41*10^-7^ |  |  |
| rdACE2 E38D | 2986.9 | 400, 200, 100, 50, 25 | 9.26*10^5^ | 1.39*10^-1^ | 1.50*10^-7^ | 2.45*10^-7^ | 6.68*10^-8^ |
|  |  |  | 6.41*10^5^ | 1.84*10^-1^ | 2.87*10^-7^ |  |  |
|  |  |  | 6.37*10^5^ | 1.89*10^-1^ | 2.97*10^-7^ |  |  |
| rdACE2 T82M | 1877.3 | 400, 200, 100, 50, 25 | 1.21*10^6^ | 1.02*10^-1^ | 8.44*10^-8^ | 9.08*10^-8^ | 4.79*10^-9^ |
|  |  |  | 1.01*10^6^ | 9.33*10^-2^ | 9.21*10^-8^ |  |  |
|  |  |  | 1.02*10^6^ | 9.79*10^-2^ | 9.59*10^-8^ |  |  |
| rdACE2 D90N | 2747.9 | 400, 200, 100, 50, 25 | 6.59*10^5^ | 5.87*10^-1^ | 8.91*10^-7^ | 1.20*10^-6^ | 2.18*10^-7^ |
|  |  |  | 3.95*10^5^ | 5.20*10^-1^ | 1.32*10^-6^ |  |  |
|  |  |  | 3.85*10^5^ | 5.32*10^-1^ | 1.38*10^-6^ |  |  |
| rdACE2 R353K | 2653.6 | 400, 200, 100, 50, 25 | 7.62*10^5^ | 1.01*10^-1^ | 1.33*10^-7^ | 1.36*10^-7^ | 2.76*10^-9^ |
|  |  |  | 7.06*10^5^ | 9.67*10^-2^ | 1.37*10^-7^ |  |  |
|  |  |  | 3.64*10^6^ | 5.07*10^-1^ | 1.39*10^-7^ |  |  |
| hACE2 | 6150.5 | 400, 200, 100, 50, 25 | 2.97*10^5^ | 4.20*10^-3^ | 1.41*10^-8^ | 1.23*10^-8^ | 1.32*10^-9^ |
|  |  |  | 3.16*10^5^ | 3.63*10^-3^ | 1.15*10^-8^ |  |  |
|  |  |  | 2.46*10^5^ | 2.76*10^-3^ | 1.12*10^-8^ |  |  |
