# Supplementary material for: Receptor binding and structural basis of raccoon dog ACE2 binding to SARS-CoV-2 prototype and its variants
Source: PLoS Pathog. 2024 Dec 5;20(12):e1012713. doi: 10.1371/journal.ppat.1012713 (PMC11620640; doi:10.1371/journal.ppat.1012713)
Supplement: S7 Table — (DOCX) [file ppat.1012713.s013.docx]

**Table S7** The immobilization and concentrations statistics of SPR assay to test the binding affinities between ACE2 and PT K417N RBD

| **Ligand** | **Immobilization quantity (units)** | **Concentrations of RBD**  **(nM)** | ***k*_a_ (1/Ms)** | ***k*_d_ (1/s)** | ***K*_D_ (M)** | **Average *K*_D_ (M)** | **SD  (M)** |
| --- | --- | --- | --- | --- | --- | --- | --- |
| rdACE2 | 2621.0 | 200, 100, 50, 25, 12.5 | 1.69*10^5^ | 5.27*10^-2^ | 3.11*10^-7^ | 4.70*10^-7^ | 1.49*10^-7^ |
|  |  |  | 1.13*10^5^ | 4.87*10^-2^ | 4.30*10^-7^ |  |  |
|  |  |  | 8.26*10^4^ | 5.52*10^-2^ | 6.68*10^-7^ |  |  |
| rdACE2 L24Q | 2738.0 | 200, 100, 50, 25, 12.5 | 1.42*10^5^ | 1.34*10^-2^ | 9.45*10^-8^ | 1.53*10^-7^ | 4.97*10^-8^ |
|  |  |  | 8.80*10^4^ | 1.31*10^-2^ | 1.48*10^-7^ |  |  |
|  |  |  | 8.30*10^4^ | 1.79*10^-2^ | 2.16*10^-7^ |  |  |
| rdACE2 Y34H | 3120.9 | 200, 100, 50, 25, 12.5 | 1.77*10^5^ | 6.50*10^-3^ | 3.68*10^-8^ | 5.50*10^-8^ | 1.62*10^-8^ |
|  |  |  | 1.12*10^5^ | 5.83*10^-3^ | 5.20*10^-8^ |  |  |
|  |  |  | 8.79*10^4^ | 6.69*10^-3^ | 7.61*10^-8^ |  |  |
| rdACE2 E38D | 2986.9 | 200, 100, 50, 25, 12.5 | 1.35*10^5^ | 1.31*10^-2^ | 9.74*10^-8^ | 1.13*10^-7^ | 2.91*10^-8^ |
|  |  |  | 1.05*10^5^ | 8.88*10^-3^ | 8.69*10^-8^ |  |  |
|  |  |  | 8.27*10^4^ | 1.27*10^-2^ | 1.53*10^-7^ |  |  |
| rdACE2 T82M | 1877.3 | 200, 100, 50, 25, 12.5 | 1.23*10^5^ | 4.64*10^-3^ | 3.78*10^-8^ | 4.92*10^-8^ | 8.15*10^-9^ |
|  |  |  | 1.09*10^5^ | 5.81*10^-3^ | 5.32*10^-8^ |  |  |
|  |  |  | 9.93*10^4^ | 5.62*10^-3^ | 5.66*10^-8^ |  |  |
| rdACE2 D90N | 2747.9 | 200, 100, 50, 25, 12.5 | 4.49*10^3^ | 2.99*10^-1^ | 6.67*10^-5^ | 6.14*10^-5^ | 2.21*10^-5^ |
|  |  |  | 7.08*10^3^ | 2.27*10^-1^ | 3.21*10^-5^ |  |  |
|  |  |  | 1.99*10^3^ | 1.70*10^-1^ | 8.55*10^-5^ |  |  |
| rdACE2 R353K | 2653.6 | 200, 100, 50, 25, 12.5 | 1.39*10^5^ | 8.45*10^-3^ | 6.08*10^-8^ | 1.05*10^-7^ | 3.12*10^-8^ |
|  |  |  | 8.29*10^4^ | 1.02*10^-2^ | 1.23*10^-7^ |  |  |
|  |  |  | 8.13*10^4^ | 1.06*10^-2^ | 1.30*10^-7^ |  |  |
| hACE2 | 6150.5 | 400, 200, 100, 50, 25 | 3.78*10^5^ | 1.40*10^-2^ | 3.71*10^-8^ | 4.71*10^-8^ | 7.73*10^-9^ |
|  |  |  | 3.05*10^5^ | 1.47*10^-2^ | 4.81*10^-8^ |  |  |
|  |  |  | 3.36*10^5^ | 1.88*10^-2^ | 5.59*10^-8^ |  |  |
